# Highlights from Heart Rhythm Society 2017: Introductory Comments

**DOI:** 10.19102/icrm.2017.080807

**Published:** 2017-08-15

**Authors:** Brian Olshansky


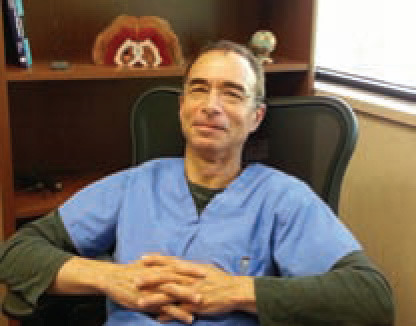


The recent Heart Rhythm Society (HRS) 2017 meeting was a remarkable success and definitely worth attending. More than 900 faculty members contributed to over 250 educational sessions, covering almost every conceivable arrhythmia topic available, including pacing, defibrillation, arrhythmia management, ablation, pharmacology, genetics, basic science, health policy, and autonomics, and at all content levels, from that for the novice physicians and electrophysiology (EP) fellows to that for experienced nurses, nurse practitioners, and basic and clinical electrophysiologists. Various original studies, some of the best new research in the field, were also presented. The depth and breadth of the meeting is hard to capture and distill down to just a few topics or presentations—there were simply too many to attend, with some being simultaneously ongoing competitive sessions, to catch a glimpse of all that was important. One good place to start, perhaps, is the late-breaking clinical trials, but to focus on these alone does not do justice to the numerous sessions that comprised the meeting.

## Above and beyond

Because, recently, other meetings in the United States have been struggling, one may wonder why HRS 2017 was so diverse, energetic, poignant, and full of innovation. One answer is that HRS is an international organization with a broad reach. The diversity of membership is one of its major strengths, and its focus is on providing the best new science and education to young and old individuals alike. Rarely is there an organization that has as its members practicing physicians, basic scientists, trainees, industry representatives, and associated professionals, who all work together in a collaborative way. The attendance for this year’s meeting was impressive, and the depth of knowledge presented extensive, making this one of the premier EP meetings. There was a wide range of topics discussed; excellent reviews in all critical areas of arrhythmia management shared; and new data presented in many forms, including the late-breaking clinical trials, poster presentations, and oral abstracts and review sessions.

It would be an injustice to focus only on certain specific trials or presentations as being items that could potentially change the clinical practice of EP because the scope of the 2017 show was so immense. I will instead focus on some of the events that I had an opportunity to see and that I thought added substantially to the practice of EP. My experience may not reflect all critical presentations as, due to the size of the meeting, it was impossible to attend all sessions. As I was involved specifically in several such sessions, I think it is worth mentioning that for those who were unable to attend portions of the meeting outside of the late-breaking clinical trials, much information of interest was also presented.

Superb sessions included expert reviews on multiple topics. I was involved and/or attended several of these sessions, which included presentations on the use of different imaging modalities—including MRI, PET, and CT scans and the like—in terms of assisting with prognostication and therapeutic intervention in patients with potentially life-threatening and symptomatic arrhythmias. One superb and well-rounded session focused on tachycardia-induced cardiomyopathy, and presented an understanding of the basic mechanisms, conditions of importance, and therapeutic interventions. An arrhythmia response session concerning athletes and arrhythmias also proved interesting: not only was new information presented, but also the qualifications of the audience to interpret and intervene on arrhythmias in athletes that were divulged were enlightening. Another session involving the sharing of novel research concerning how to define high-risk populations for implantable cardioverter-defibrillator (ICD) therapy and proper programming was also valuable. A session on the role of implantable devices in special populations, where attendees had the opportunity to experience a wide range of important clinical topics, provided unique and practical information for patient management.

Several key developments are worth considering. The use of monitoring is a growing and important issue, especially its proper use in patients with atrial fibrillation (AF) who are at high risk but who are minimally symptomatic. New uses for implantable loop recorders continue to be uncovered, and there are major advancements underway in the technology as a whole, and continued improvements being made in external loop recording specifically. It is believed that remote monitoring has a growing role in the care of patients. This is particularly noteworthy for its potential as an intervention to offset long-term risks, and for prognostication with novel parameters such as heart rate score.

Novel information was presented about vascular occlusion during lead extraction to prevent exsanguination. This appears to have an important role in select patients, and may even be life saving. Likely, it will become an important tool to reduce the risk of adverse outcomes associated with the procedure. However, like the role of an intranasal drug to stop supraventricular tachycardia, the role will likely be limited.

Remarkable advances in device technology, including breakthroughs in the use of subcutaneous ICDs and leadless pacemakers, are upon us. Some of this information, presented at the late breaking clinical trials, was interesting and the innovations may eventually become game changing. There is a growing need for better pacemakers and defibrillators that are effective, and those that can be implanted with minimal risk. Perhaps the release of subcutaneous devices capable of providing biventricular pacing is not that far off. Such developments are part of work in progress, about which we will no doubt hear at future meetings.

However, perhaps one day, we will even abandon biventricular pacing for other options. Another point of note from this year’s HRS meeting was the growing interest in His-bundle pacing. First and foremost, it works! Secondly, there appears to be a growing role for its use in patients with ventricular dysfunction and left bundle branch block.

State-of-the-art lectures on ventricular tachycardia and AF were presented. Regarding AF ablation, despite all of the extensive investigations that have been conducted to date, the cure of persistent AF remains an unknown and unattainable concept. A new document, recently released, and discussed this year, titled “2017 HRS/EHRA/ECAS/APHRS/SOLAECE Expert Consensus Statement on Catheter and Surgical Ablation of Atrial Fibrillation,” encapsulates what is known currently in the fast-moving field of AF ablation.

Improved mapping may facilitate more effective treatment of complex atrial and ventricular arrhythmias. Fortunately, advances in electroanatomical mapping to understand the mechanism of and ablate complex arrhythmias are upon us. New mapping techniques continue to be developed. CARTO^®^ (BioSense Webster, Diamond Bar, CA, USA) is one such technology; Rhythmia™ (Boston Scientific, Natick, MA, USA) is another. No doubt, better mapping systems will continue to be developed as technology advances further. I look forward to seeing data showing improved outcomes with better technology, but such does raise the issue of the collaboration of industry with medical science, and how exactly that should be done to yield the best outcome possible. At one of the first HRS meetings I attended in the 1980s (known as NASPE at the time), all of the technology that was there was presented on one small table in one small room. Consider how far we have come in 35 years!

## Moving forward

Returning to thinking about the current meeting, there were advancements in knowledge presented concerning arrhythmia mechanisms, specifically with regards to the better understanding of the relationship of obesity and sleep apnea and AF. There was even data presented on the use of a short-acting drug to stop supraventricular tachycardia by an intranasal route.

Attempts to utilize big data to better understand therapies and how they affect the outcomes of patients with arrhythmias continue. This approach, and the combination of smaller data sets into a single larger one, is becoming more the norm, even though the conclusions that can be drawn may remain seriously limited. As someone who has been involved in using such data, I am aware that major conclusions can be drawn from “real life” but non-randomized and retrospective data. We need to use this information with care.

Thus, HRS 2017 left every participant with new information, new ideas, and new excitement. I look forward to specific comments from my colleagues regarding what struck them as most important, and of course, I look forward to HRS 2018.

Brian Olshansky, MD, FHRS, FACC, FAHA

E-mail: brian-olshansky@uiowa.edu

Professor Emeritus

University of Iowa

Cardiac Electrophysiologist

Mercy Hospital-North Iowa

Mason City, Iowa 50401

